# A Study of the Response Surface Methodology and Finite Element Method to Model Aircraft Engine Body Deformations During Hole Drilling

**DOI:** 10.3390/ma17246214

**Published:** 2024-12-19

**Authors:** Andrzej Matras, Magdalena Machno

**Affiliations:** 1 Department of Production Engineering, Faculty of Mechanical Engineering, Cracow University of Technology, 31-155 Cracow, Poland; 2 Department of Rail Vehicles and Transport, Faculty of Mechanical Engineering, Cracow University of Technology, 31-155 Cracow, Poland; magdalena.machno@pk.edu.pl

**Keywords:** Inconel 718, drilling, finite element method, deformation, aircraft engine body

## Abstract

The aviation industry is still looking for effective manufacturing methods. Currently, the challenge is still the machining of shapes in thin-walled materials. This work focuses on the analysis of the influence of these parameters on deformations during the drilling process of holes in the adapters of aircraft engine body accessories. The drilling process was carried out in the thin-walled superalloy Inconel 718, which is classified as a difficult-to-machine material. During the analyses, experimental studies based on the Response Surface Method (RSM) and finite element method (FEM) calculations were carried out simultaneously. The aim of this work was to analyze the developed mathematical models describing nonlinear relationships between cutting parameters, cutting forces, and deformations of the aircraft engine body characterized by a complex, thin-walled geometric structure. By using the proposed solutions, it is possible to achieve integration between the techniques of conducting research, performing calculations using FEM, and designing the machining. The advantage of this comprehensive approach used in our work is the development of mathematical models that strongly fit the results of the research. The results of analyses and calculations presented in the article and the research methodology used can be applied to industrial conditions.

## 1. Introduction

In the aerospace industry, more and more parts are manufactured with complex geometry. These parts are also made of durable materials with specific thermophysical properties. Among the most commonly used materials for these parts are nickel–chromium superalloys. One of the most frequently used superalloys for the production of parts in the aerospace industry is Inconel 718 [1].

The use of the Inconel 718 superalloy for parts in the aerospace industry is due to its very good strength properties, work hardening, the presence of highly abrasive carbide particles, high toughness, high hardness, and poor thermal properties [2,3]. However, these properties of Inconel 718 make its machining difficult; hence, its machining is a challenge for conventional methods. Therefore, this superalloy is classified as part of difficult-to-machine materials [4,5]. The strength of Inconel 718 at elevated temperatures is quite high. This means that its machining requires the presence of a high cutting force for effective machining. This contributes to the delivery of a huge amount of heat to the tool tip. Moreover, during the machining process, the removed material has a strong tendency to adhere to the tool and form built-up edges on the cutting edge. As a consequence, the tool wears out faster, and the workpiece is plastically deformed [2,3,6].

The properties of the difficult-to-cut Inconel 718 superalloy cause a challenge for drilling holes. There are many works in the literature that describe the challenges of drilling holes in this type of advanced material. Ahmed et al. [7] analyzed the process of drilling holes in a thin-walled gun barrel made of Inconel 718 alloy. They developed a mechanistic model by taking into account force, drill deflection, hole wall deformation, and process kinematics, supported by experimental results. By modifying the cutting parameters, they achieved an improvement in the geometry of the holes. Zhang et al. [8] investigated the process of drilling holes in thin-walled plates. Using the finite element method and experimental studies, they determined the effect of temperature on the degree of damage to the hole geometry. Biruk-Urban et al. [9] investigated the influence of cutting parameters on the force values and quality of holes made in thin-walled composite plates. They stated that in order to obtain high hole quality and improve the drilling process, it is important to monitor and control the cutting forces during drilling. They also showed that feed is one of the most important parameters influencing hole quality. Szwajka et al. [10] analyzed the process of drilling holes in plates made of the Inconel 625 alloy. In order to reduce the value of forces, they proposed the use of pre-treatment using a laser beam. As a result of the reduction in cutting forces, deformations were reduced. Luo et al. [11] investigated the relationship between force and deformation during the drilling of low-stiffness CFRP/Ti stacks. They developed a model for predicting the cutting force. They determined the hole damage factor and the effect of feed on its value. In future work, it is planned to use the finite element method (FEM) for this purpose.

In addition, in order to better understand and visualize the influence of process parameters on the analyzed outcome factors, various approximation methods can be used. These methods are used to optimize the data [12]. Among the most commonly used mathematical and statistical techniques, we can distinguish factors such as the multiple regression and design of experiments (DOEs) [13], THE Response Surface Methodology (RSM) [14], Tauguchi method [15], Gray relational analysis (GRA) [16], Artificial Neural Networks (ANNs) and neuro-fuzzy approach [17], as well as a combination of these techniques [18]. One of the frequently used techniques is RSM. The RSM method includes a set of mathematical and experimental techniques that require a sufficient number of experimental data to analyze the problem and develop mathematical models that take into account the relationships of several input variables and result factors [19]. This feature is important because, in machining processes, the influence of many process factors on the resulting factors (usually three, four, or more) is often analyzed [12]. In addition, for a more detailed understanding of the relationships of the process factors for subsequent data optimization, FEM is also used [20].

The above analysis shows that different methods are used to analyze data and the influence of machining parameters in the cutting process. The FEM can be effectively used to calculate the deflections of thin-walled elements characterized by a complex geometric structure. Gameros et al. [21] investigated the deformation of thin-walled parts with a complex geometric structure that occurred during their machining. The calculations performed using FEM were verified by real measurements. They obtained differences in the results in the range of 4.04%. However, in [22], the deformation of a car steering knuckle with a complex geometric structure was investigated. Using FEM, the model with forces occurring in the machining process was loaded. As a result of the verification of the calculations, depending on the applied load, discrepancies were obtained, ranging from 6.8 to 17.3%. Dharmavaram Narendranath et al. [23] analyzed the deflection of a thin-walled object. As a result of using the finite element method for calculations, depending on the element thickness, they obtained a maximum error in the range of 15.2%. The analysis of the above works shows that FEM is a frequently used method for the analysis of forces occurring in the machining process. This is also the reason why this method was used in this work to analyze the results of the abrasion. However, in this work, we decided to combine it with RSM.

According to the conducted data analysis and conclusions from the experience of machining thin-walled parts made with the Inconel 718 superalloy, effective machining methods are still needed. Many difficulties and challenges are still encountered in the effective production of shapes in thin-walled surfaces of parts used in the aerospace industry. The difficulties result from the specific properties of Inconel 718 and the geometry of the machined material (thin-walled material). As the literature shows, more and more data analysis programs are being used to better understand the effect of process parameters, such as RSM, DOE, and ANNs. CAD/CAM (Computer-Aided Design/Computer-Aided Manufacturing) engineering support systems are also helpful.

The above analysis of scientific papers shows that different data optimization methods can be used to analyze the results. This shows that a strong fit of mathematical models to the results from the research and measured results in machining processes is still a challenge. New methods for analyzing the results are also still being developed. It is important that the method used provides a strong fit for the developed mathematical models. Currently, the results of machining research are more difficult to analyze due to the machining of modern engineering materials, which often gives ambiguous results. There is still a gap in the issue of using an appropriate method to analyze the results from the machining of modern superalloys. An additional difficulty is the geometry of the machined material, e.g., thin-walled parts and drilling holes in them.

In this work, an analysis of the process of drilling holes in equipment adapters of an aircraft engine body made of Inconel 718 was performed. The adapters are located on the thin-walled geometry of the body. The analysis of the relationship between input and output factors was performed using the RSM. In addition, the body deflection and hole axes displacement calculations were performed using the FEM. The unique combination of these two techniques allowed us to determine the influence of cutting parameters used in the drilling process on the deformation of the aircraft engine body, which is extremely important in the process of machining design. Moreover, both techniques are effective tools for modern engineers and can be successfully used in the real process of aircraft engine body machining design. The research methodology proposed in the article does not require the use of a large number of expensive tests and does not require a large consumption time. In this way, the integration between experimental studies, experiment design techniques, FEM calculations, and the machining design process in CAD/CAM (Computer-Aided Desing/Manufacturing) systems has also been achieved. Based on the proposed solution, the correction factors of the individual hole’s positions and the stability conditions for thin-walled aircraft engine body holes during the drilling process can be determined for the real machining design process.

This article was prepared in the following order, which was adopted in the development of the research material:

- Chapter 2 describes in detail the applied research methodology and the method of performing experiments and analyses.
- The Results section includes the following points:

1. The influence of cutting parameters on forces and moments;
2. The analysis of the influence of cutting parameters on the total engine body deflection;
3. The influence of cutting parameters on radial displacements of the hole axes.

The paper also includes a Discussion (which discusses the results of the conducted research) and a Conclusions section (which summarizes the article by presenting the most important findings).

## 2. Materials and Methods

The Inconel 718 superalloy was selected as the workpiece material for the experimental tests. Inconel 718 is widely used in the aerospace industry, including thin-walled aircraft engine bodies, biomedicine, and advanced automotive industries. Such a wide application of this superalloy and the challenges associated with its machining determined its selection for in-work tests. The chemical composition of the used Inconel 718 superalloy is shown in Table 1.

**Figure 1:**
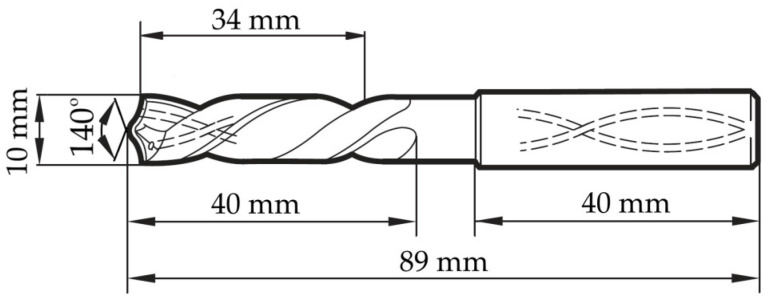
Geometry of the drilling tool.

Holes were drilled into the test material using a 10 mm diameter Gühring RT100F carbide drill (Gühring KG, Albstadt, Germany). The used drill has two symmetrical cutting edges and a TiN coating. It is characterized by a short type of construction and is manufactured in accordance with the DIN 6537 K standard. The drill can be used to make holes with a maximum length of 30 mm. The processed material had a thickness of 20 mm. During machining, the cutting zone was lubricated using the Minimum Quantity Lubrication (MQL) method. Figure 1 shows a scheme of the drill with its geometric parameters.

**Figure 2:**
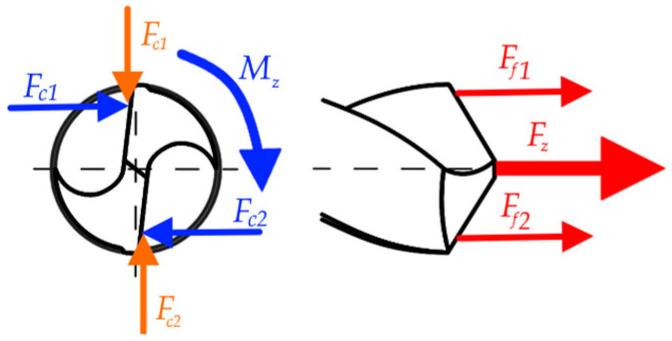
The system of forces occurring in the drilling process.

The aim of the experimental study was to determine the effect of the cutting speed *v_c_* and the feed per revolution *f* on the values of the axial component of the cutting force *F_z_* and the torque measured in the tool axis *M_z_*. The system of forces occurring in the analyzed drilling process is shown in Figure 2. The axial force *F_z_* is the sum of the feed forces *F_f_*_1_ and *F_f_*_2_. The moment *M_z_* was obtained from the forces *F_c_*_1_ and *F_c_*_2_. The forces *F_p_*_1_ and *F_p_*_2_ reduce each other, so there was no resultant force.

**Figure 3:**
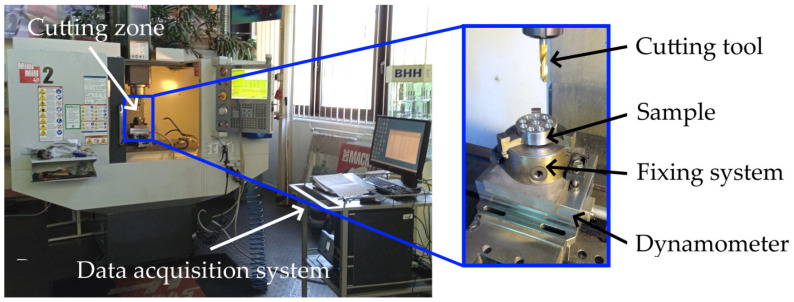
Experimental test stand.

To measure the cutting force, a measuring stand was set up based on the numerically controlled MiniMill2 machine (Haas Automation, Oxnard, CA, USA), the Kistler 9257B force gauge, the Kistler 5070A charge amplifier, and the Kistler DynaWare software (Kistler Instrumente AG, Winterthur, Switzerland). The 9257B piezoelectric force gauge converts forces and moments into a proportional electrical signal. This signal is amplified by a 5070A amplifier, converted to digital format, and analyzed in DynaWare v2825A software. The total measurement uncertainty of the equipment for measuring the force components is 3.6% FSO. The test stand is shown in Figure 3.

In addition to the experimental research, calculations were also performed using the Finite Element Method (FEM). The aim of the research conducted was to determine the influence of forces and moments measured in the first stage on the engine body deflection. The maximum body deflection and the displacement of the axis of the currently drilled hole measured in the radial direction were analyzed. The displacement of the hole axis in the axial direction was not analyzed.

The studies were conducted based on a central compositional plan. The choice of the central compositional plan resulted from the use of the Response Surface Method. Two variables were analyzed at five levels. The values of the analyzed cutting parameters were determined based on the recommendations of the cutting tool manufacturer. The ranges of the variation in the cutting parameters calculated for the applied research plan were 0.065–0.135 mm/rev for feed *f* and 17.93–32.07 m/min for cutting speed *v_c_*, respectively. The analyses of the measurements and calculations results were performed in the Statistica software v13.3 (TIBCO Software Inc., Palo Alto, CA, USA) for the assumed significance level of α = 0.05. Table 2 shows the applied research plan together with the tested cutting parameter values and the order in which the tests were performed.

During the measurements, the force and moment waveforms were recorded when the hole was drilled. Then, the mean value of the waveform was determined. For each test in the research plan, three holes and three force and moment measurements were performed. The stages of the plunge and exit of the drill from the material were not analyzed during the measurements. The signal sampling rate was set to 10 kHz.

**Figure 4:**
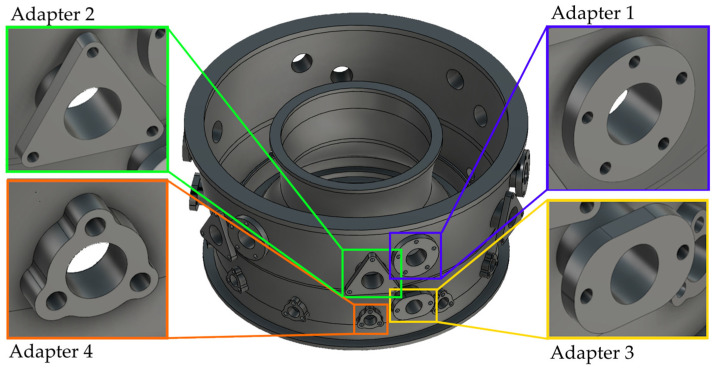
Designed model of the aircraft engine body with a view of the adapters.

For the purpose of conducting research and analysis, an aircraft engine body was designed with four types of tooling adapters located in different places on the body (Figure 4). The largest external diameter of the body was 780 mm, while the height of the body was 390 mm. All holes in the tooling adapters were through holes. Figure 4 shows a view of the designed body and views of all types of tooling adapters.

Based on experimental data and the results of FEM, mathematical models were developed using RSM. The mathematical modeling aimed to check the effect of cutting parameter values (*v_c_*, *f*) on the force *F_z_* and cutting torque *M_z_*, as well as the effect on the aircraft engine body’s deflection occurring during drilling holes. Using the RSM method, the mathematical model describes the relationship between the process parameters and the analyzed result factors, which is described by Equation (1):

(1) $$Y=f\left(v_{c},f\right)\pm \varepsilon ,$$

where *Y* is the desired response, *f* is the response function (or response surface), and *ε* is the fitting error.

In the analysis procedure, the approximation of *Y* was proposed by using the fitted second-order polynomial regression model, also called the quadratic model. This model (the research object function) can be written as follows by Equation (2):

(2) $$Y=\beta_{0}+\sum_{i=1}^{k}\beta_{i}x_{i}+\sum_{i=1}^{k}\beta_{ii}x_{i}^{2}+\sum_{i<j}^{k}\beta_{ij}x_{i}x_{j}\pm \varepsilon ,$$

where *k* is the number of input process parameters, *x_i_* corresponds to the input variables, *x_i_*^2^ and *x_i_x_j_* are the squares and interaction terms, respectively, *β*_0_ is a constant, and *β_i_*, *β_ii_*, and *β_ij_* represent the coefficients of linear, quadratic, and cross-product terms, respectively [1].

The evaluation of the obtained models was performed based on the calculation of the Pearson correlation coefficients *R* (Equation (3) and analysis of Predicted vs. Observed plots.

(3) $$R=\frac{\sum_{i=1}^{N}\left(E_{i}-\bar{E}\right)\cdot \left(O_{i}-\bar{O}\right)}{\sqrt{\sum_{i=1}^{N}{\left(E_{i}-\bar{E}\right)}^{2}}\cdot \sqrt{\sum_{i=1}^{N}{\left(O_{i}-\bar{O}\right)}^{2}}}$$

where

*N*—the number of observations;
*E_i_*—the predicted value of case *i*;
*Ē*—the mean of the predicted values;
*O_i_*—the observed value of case *i*;
*Ō*—the mean of the observed values.

The Fusion360 v2.0.20981 software and Autodesk Nastran 19.0.0.1 solver (Autodesk, Inc., San Rafael, CA, USA) were used to design the engine case’s body geometry and were calculated using FEM.

The calculations were performed based on a predefined material model of the Inconel 718 superalloy. The parameters of the applied material model are listed in Table 3.

The FEM tests were divided into two stages. The first preliminary stage was performed to exclude inactive fragments of geometry from further analyses. This operation allowed for the analysis of less complex geometry, which made the calculations faster. During this stage, one of the holes in the adapter was loaded with the maximum value of the force and moment recorded during the performed measurements. The loads were applied to the cylindrical surface of the hole. Then, the maximum deflection calculation was performed. After performing the calculations, the procedure was repeated for the remaining adapters. As a result of the calculations performed, a fragment of the geometry for which no deflection was observed in each of the analyzed cases was excluded from future calculations. In order to minimize the number of mesh nodes, holes were also plugged, except for the currently analyzed tooling adapter hole.

In the second stage, which is the proper stage of FEM calculations, one hole was also loaded simultaneously. The force and moment values used to load the model were calculated as the arithmetic mean of the three force and moment measurements. Only one selected hole in each adapter was analyzed. After the FEM calculations for all points in the research plan, the next adapter was analyzed.

**Figure 5:**
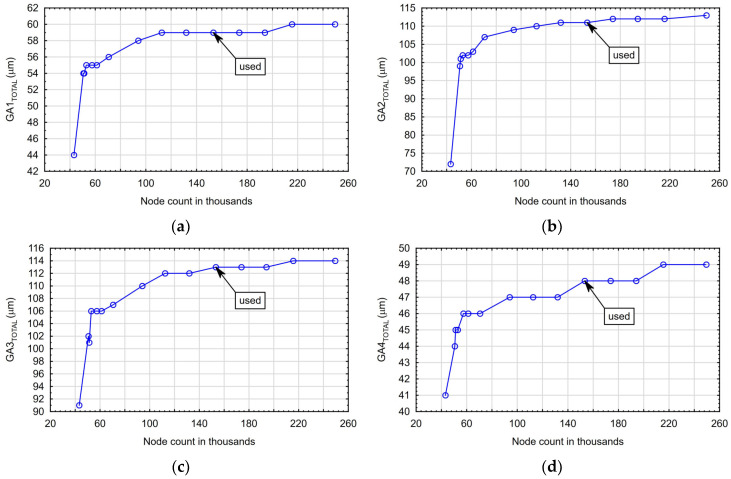
The mesh convergence graph for the body deformation *GA_TOTAL_* occurring during the drilling of the holes in adapters: (**a**) *GA1_TOTAL_*; (**b**) *GA2_TOTAL_*; (**c**) *GA3_TOTAL_*; and (**d**) *GA4_TOTAL_*.

For all FEM calculations, the finite element mesh was built based on the triangle method, and dynamic mesh scaling was based on the model element sizes, which was 3%. The finite element meshes created on the simplified models (for the second-stage calculations) contained about 165 thousand nodes and 86 thousand elements. The mesh convergence graph for the body deformation *GA_TOTAL_* occurring during the drilling of the hole in individual adapters is shown in Figure 5a–d.

**Figure 6:**
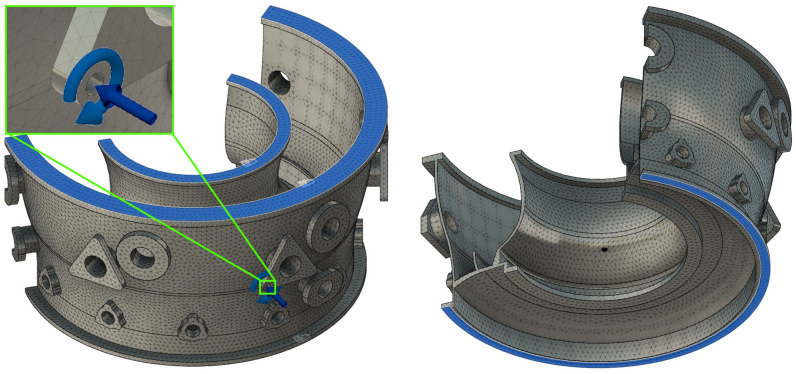
Isometric views of a simplified aircraft engine body with finite element mesh, restraints, and applied loads.

In order to reflect the method of fixing the parts during hole machining, on three flanges marked in blue (as seen in Figure 6), restraints were defined that blocked translations and rotations around all axes. Figure 6 shows isometric views of the simplified body together with the finite element mesh, fixings, and applied loads.

**Figure 7:**
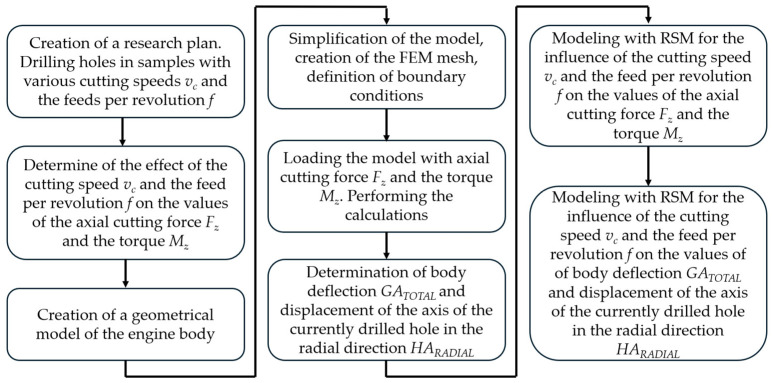
Flow chart for the applied research methodology.

The research methodology described is presented in the form of a flow chart in Figure 7.

## 3. Results

### 3.1. Influence of Parameters on Forces and Moments

**Figure 8:**
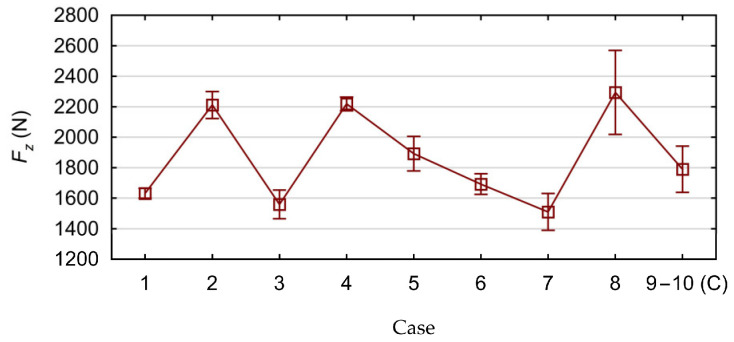
Measured axial force values with standard deviations.

**Figure 9:**
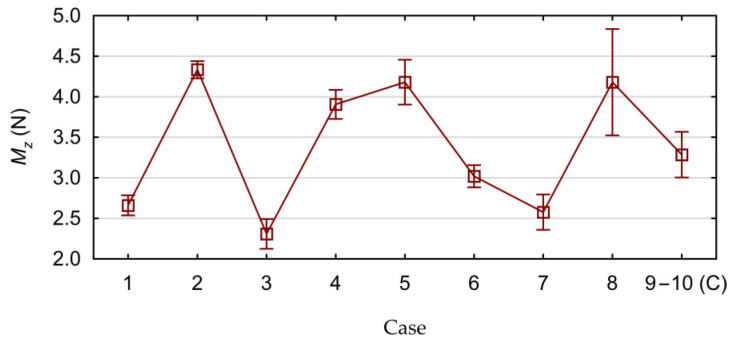
Measured cutting torque values with standard deviations.

Based on the results obtained from the experimental tests, the influence of the tested cutting parameters *v_c_* and *f* on the values of the force *F_z_* and the cutting torque *M_z_* was determined. The calculated average values of the axial force *F_z_*, together with their standard deviations, are shown in Figure 8. The calculated average values of the cutting torque *M_z_*, together with their standard deviations, are shown in Figure 9. The calculated average values are the arithmetic mean calculated from three measurements performed for each position of the research plan (Table 2).

In the first stage, ANOVA (Analysis of Variance) was performed. This type of analysis is often used to analyze the results of the cutting process. The results of the analysis are presented in Table 4, and the factors with a statistically significant influence on the measurement results are marked in bold (*p*-Value < α).

Based on the results of the performed ANOVA, it is also possible to identify factors or their interactions that have a statistically significant effect on the body deformation and the displacement of the hole axes. This assumption is based on the relationship between cutting parameters, cutting forces, and moments and body deformations. If any of the analyzed cutting parameters or their interactions do not affect the axial forces and moments simultaneously, it also cannot affect the calculated body deformations and the hole axis displacements. Therefore, based on the ANOVA performed, statistically significant factors are also determined for the models describing the body deformations and the displacements of the hole axes. Similarly to the work in Ref [24], the use of the ANOVA method proved to be appropriate and helpful for further analyses.

**Figure 10:**
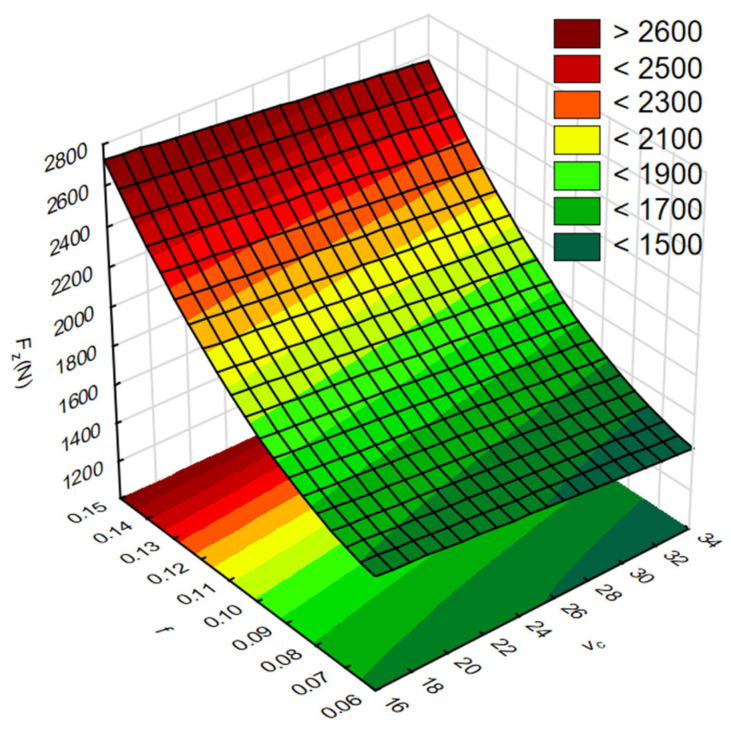
The influence of the cutting parameters *v_c_* and *f* on the force values *F_z_*.

**Figure 11:**
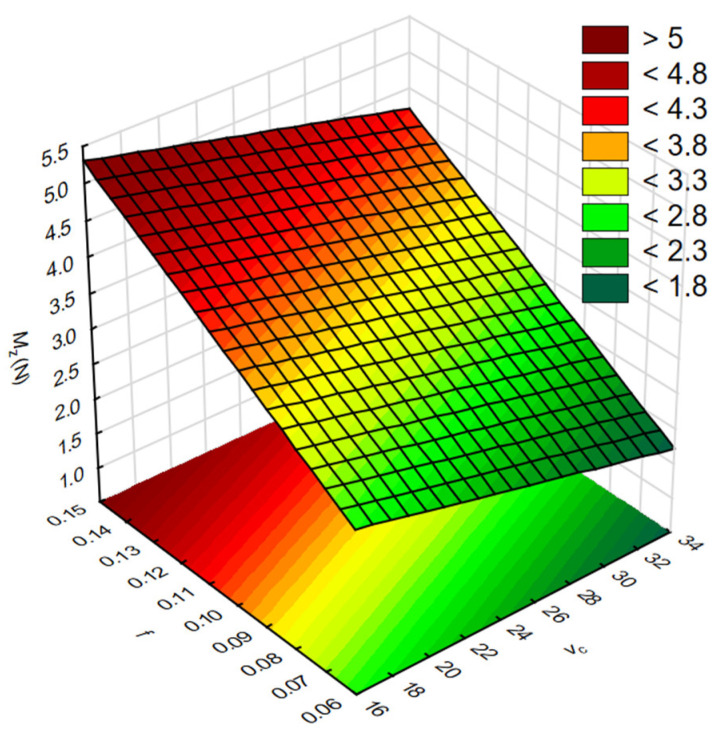
The influence of cutting parameters *v_c_* and *f* on the torque values *M_z_*.

In the next step, RSM was used to present the results of the influence of cutting parameters on the result factors. Figure 10 shows the influence of the tested cutting parameters *v_c_* and *f* on the values of force *F_z_*. In turn, Figure 11 shows the influence of *v_c_* and *f* on the values of torque *M_z_*.

The regression equations of the determined response surfaces are given below Equations (4) and (5).

(4) $$F_{z}\left(v_{c},f\right)=1860.7-8.7\cdot v_{c}-8415.7\cdot f+100746\cdot f^{2}$$

(5) $$M_{z}\left(v_{c},f\right)=2.11782-0.06055\cdot v_{c}+2.11782\cdot f$$

**Figure 12:**
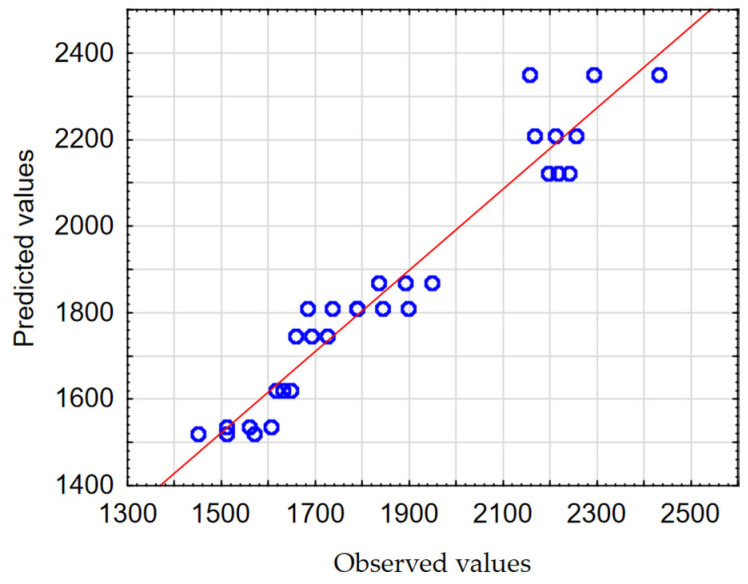
The Predicted vs. Observed plot for *F_z_*.

**Figure 13:**
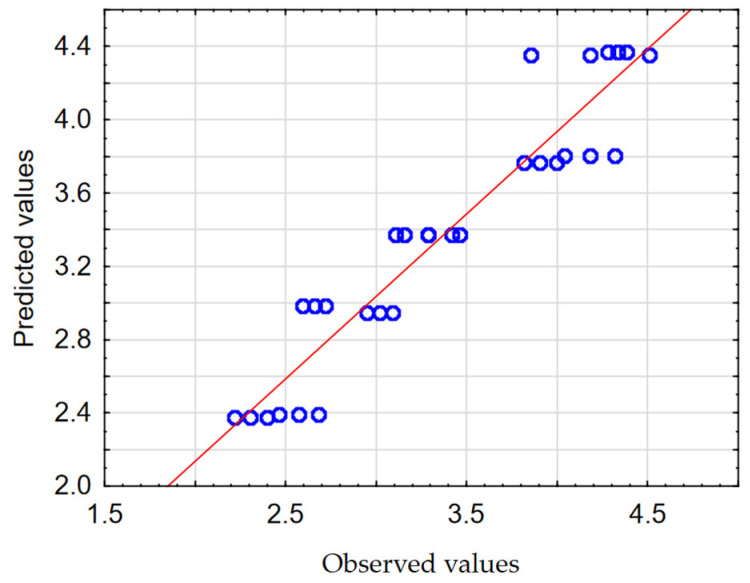
The Predicted vs. Observed plot for *M_z_*.

In order to check the quality, the obtained mathematical models were evaluated. The calculated Pearson correlation coefficients (*R*) for *F_z_* and *M_z_* were 0.968 and 0.949, respectively. The Predicted vs. Observed plots are shown in Figure 12 and Figure 13.

The results of the analysis of the influence of process parameters on the resulting factors (*F_z_*, *M_z_*) confirm the high fit of mathematical models to the experimental test results. This was confirmed, among other methods, by obtaining Pearson correlation coefficients with values close to one, and the observations are clustered around the regression line (Figure 12 and Figure 13).

### 3.2. Analysis of the Impact of Cutting Parameters on the Total Engine Body Deflection GA_TOTAL_

**Figure 14:**
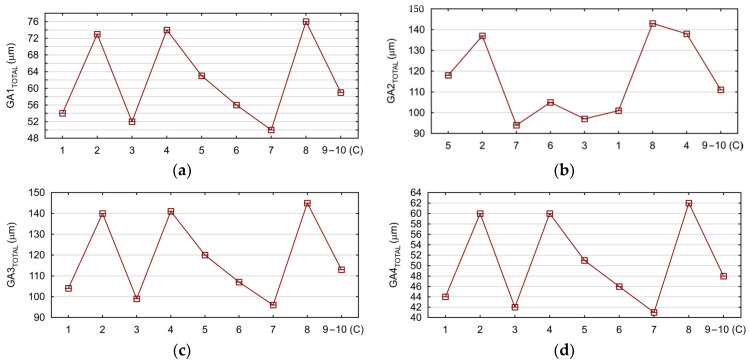
Calculated values of maximum body deflection: (**a**) *GA1_TOTAL_*; (**b**) *GA2_TOTAL_*; (**c**) *GA3_TOTAL_*; and (**d**) *GA4_TOTAL_*.

Based on the FEM calculations, the influence of cutting parameters *v_c_* and *f* on the global maximum engine body deflection values *GA_TOTAL_* was determined. The global deflection of the body values occurs when drilling holes in subsequent tooling adapters (located on adapters No. 1–4). Since there were four adapters with holes located on the analyzed body model, the analysis was performed for four adapters (*GA1_TOTAL_–GA4_TOTAL_*). The calculated deflection values are presented in Figure 14a–d.

**Figure 15:**
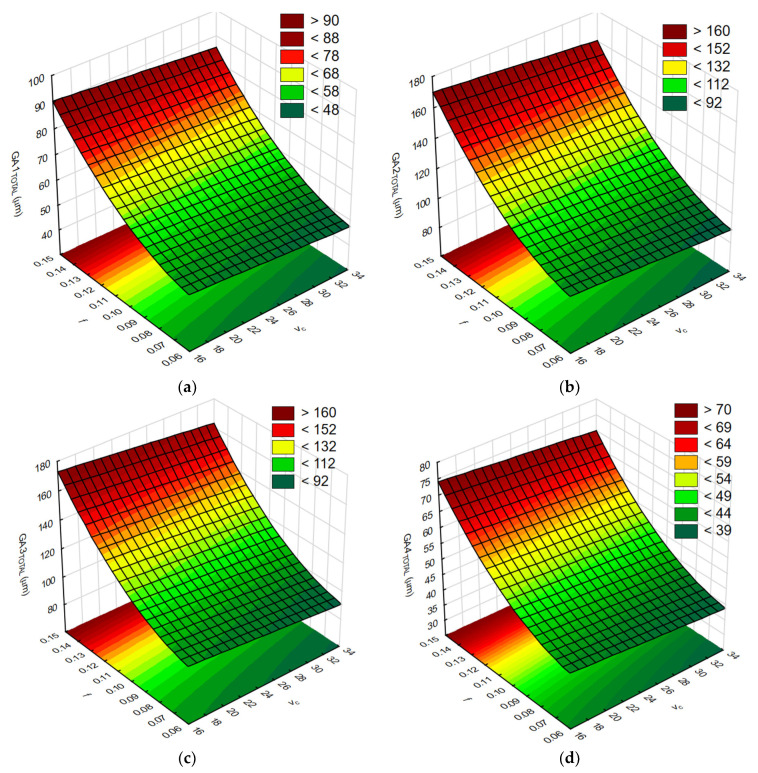
The influence of the tested cutting parameters *v_c_* and *f* on the body deflection values: (**a**) *GA1_TOTAL_*, (**b**) *GA2_TOTAL_*, (**c**) *GA3_TOTAL_*, and (**d**) *GA4_TOTAL_*. As the results show, the use of RSM to analyze the results of machining processes is a well-accepted method, similar to [14]. By analyzing the response surfaces, it is possible to determine the feed rate per revolution and the cutting speed resulting in a body deformation value that is acceptable in the machining process. The effect of feed rate on the value of body deflection is also clearly visible. Increasing the feed rate in the range below 0.1 mm/rev has a less dominant effect on body deformation than above this range. Increasing the cutting speed minimizes body deflection to a limited degree. However, this action should be considered because it has a negative effect on the cutting tool’s life. The maximum allowable load values due to possible plastic deformation of the body must also be taken into account. These values were achieved earlier for adapters for which greater deflection was observed.

In the next step, the response surfaces were determined. Figure 15a–d show the influence of the tested cutting parameters *v_c_* and *f* on the values of the body global deflection *GA_TOTAL_*.

**Figure 16:**
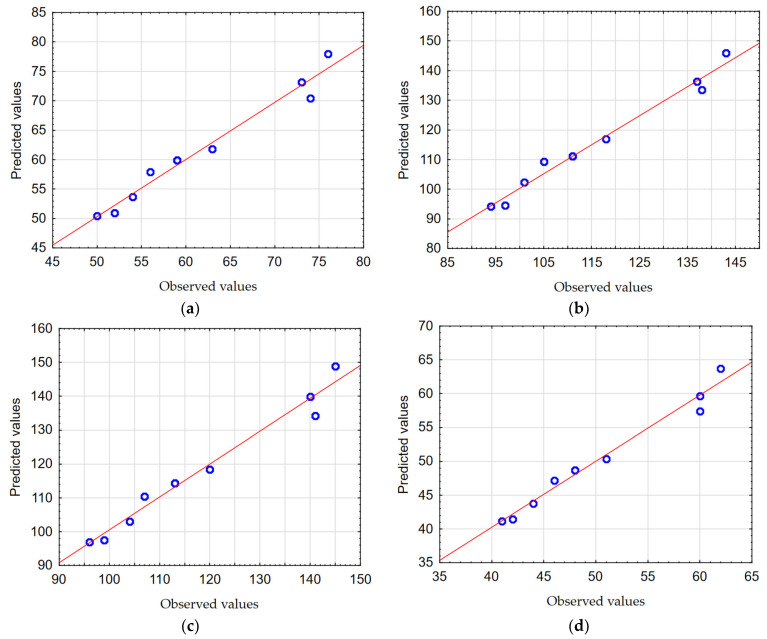
The Predicted vs. Observed plots: (**a**) *GA1_TOTAL_*, (**b**) *GA2_TOTAL_*, (**c**) *GA3_TOTAL_*, and (**d**) *GA4_TOTAL_*.

In order to check the quality, the obtained mathematical models were evaluated. The calculated Pearson correlation coefficients for *GA1_TOTAL,_ GA2_TOTAL,_ GA3_TOTAL_*, and *GA4_TOTAL_* were 0.985, 0.988, 0.986, and 0.988, respectively. The Predicted vs. Observed plots are shown in Figure 16a–d.

It was found that the mathematical models created describe the conducted experimental studies to a high degree. This was confirmed, among other methods, by obtaining Pearson correlation coefficients with values close to one and the observations are clustered around the regression line (Figure 16a–d).

### 3.3. Influence of Cutting Parameters on Radial Displacements of the Hole Axes HA_RADIAL_

**Figure 17:**
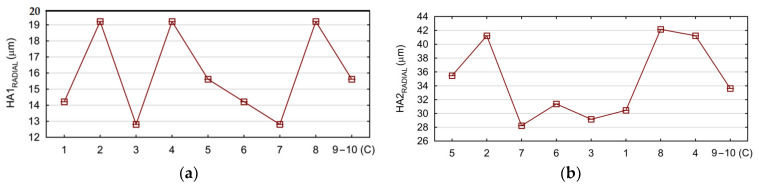

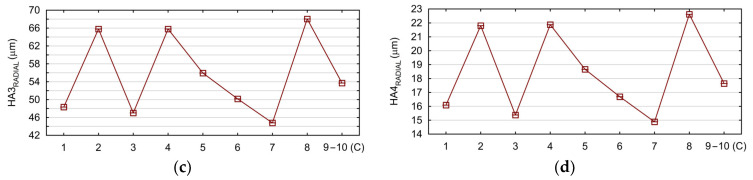
Calculated values of the displacement of the drilled hole axis in the radial direction: (**a**) *HA1_RADIAL_*; (**b**) *HA2_RADIAL_*_;_ (**c**) *HA3_RADIAL_*; and (**d**) *HA4_RADIAL_*.

In this part, the displacements of the adapter tooling hole axis on its radial direction *HA_RADIAL_* were analyzed. These analyses were performed in a similar way to the analyses of the *GA_TOTAL_* values. The analyses were performed for holes located on four adapters (*HA1_RADIAL_*, *HA2_RADIAL_*, *HA3_RADIAL_*, and *HA4_RADIAL_*). The calculated values of the hole axis displacements are presented in Figure 17a–d.

**Figure 18:**
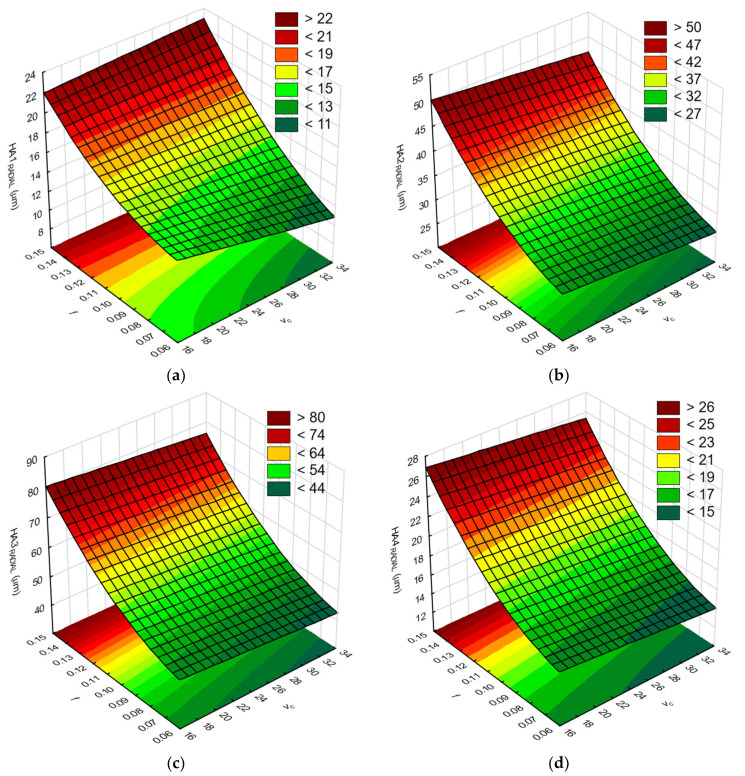
The influence of cutting parameters *v_c_* and *f* on the displacements of the drilled hole in the radial direction *HA_RADIAL_*: (**a**) *HA1_RADIAL_*; (**b**) *HA2_RADIAL_*; (**c**) *HA3_RADIAL_*; and (**d**) *HA4_RADIAL_*.

In the next step, the response surfaces were determined. Figure 18a–d show the influence of the tested cutting parameters *v_c_* and *f* on the values of the drilled hole axis displacement *HA_RADIAL_*.

**Figure 19:**
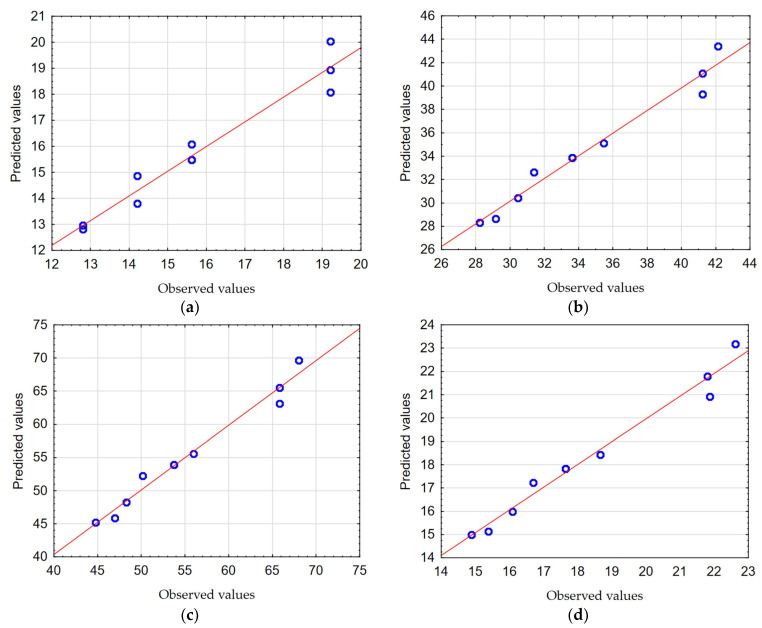
The Predicted vs. the Observed plots: (**a**) *HA1_RADIAL_*; (**b**) *HA2_RADIAL_*; (**c**) *HA3_RADIAL_*; and (**d**) *HA4_RADIAL_*.

In order to check the quality, the obtained mathematical models were evaluated. The calculated Pearson correlation coefficients for *HA1_RADIAL,_ HA2_RADIAL,_ HA3_RADIAL_*, and *HA4_RADIAL_* were 0.979, 0.985, 0.988, and 0.988, respectively. The Predicted vs. Observed plots are shown in Figure 19a–d.

It was found that the created mathematical models describe the conducted experimental studies to a high degree. This was confirmed, among other methods, by obtaining Pearson correlation coefficients with values close to one and the dates of the research are clustered around the regression line (Figure 19a–d).

## 4. Discussion

The tests allowed the influence of the analyzed cutting speeds *v_c_* and feed per revolution *f* on the values of the axial force *F_z_*, torque *M_z_*, body deflections, and hole axis displacements to be determined. The analysis of the results showed that the highest deflection values were observed on the cylindrical part of the body and on the tooling adapters. In all the analyzed cases, elastic deformations occurred. For this reason, after drilling the adapter holes, the engine case body remained undeformed. However, the deflection occurring during drilling affected the accuracy of the position of the hole being made and the inaccuracies in the geometry. In the case of the analysis of the influence of the parameters during the process of drilling holes in adapters on the cutting forces and moments, the following influences were determined:

- Increasing the range of feed per revolution *f* value causes an unfavorable nonlinear increase in the axial force *F_z_* and an unfavorable linear increase in the torque *M_z_*.
- Increasing the cutting speed *v_c_* value causes a favorable linear decrease in the values of the axial force *F_z_* and the torque *M*_z_.
- The influence of the feed *f* value is dominant on the measured values of the axial force *F_z_* and the torque *M_z_*.
- The relatively high values of the axial force *F_z_*, in the range of 1450–2432 N, and low values of the torque *M_z_* in the range of 2.22–4.51 Nm were recorded.

The relationships presented above are often observed in the process of drilling holes. Increasing the feed per revolution increases the cross-section of the cutting layer, which results in increased cutting resistance. Additionally, the volume of the material in the area of the drill’s chisel edge increased. In this area, no cutting occurred, but the material was plastically deformed and pushed out of the chisel edge. Increasing cutting resistance and the volume of plastically deformed material increases axial forces. High values of axial force are a consequence of the high mechanical properties of the processed material. Due to the plastic deformation of the material in the chisel edge work area, the axial force is dominant over the cutting torque which mainly results from cutting resistance.

**Figure 20:**
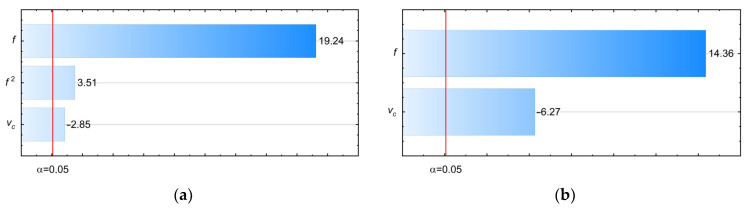
The influence of the analyzed variables on the measured values: (**a**) axial force *F_z_*; (**b**) torque *M_z_*.

In Figure 20a,b Pareto diagrams present the hierarchy of the influence of the analyzed variables on the *F_z_* and *M_z_*.

**Figure 21:**
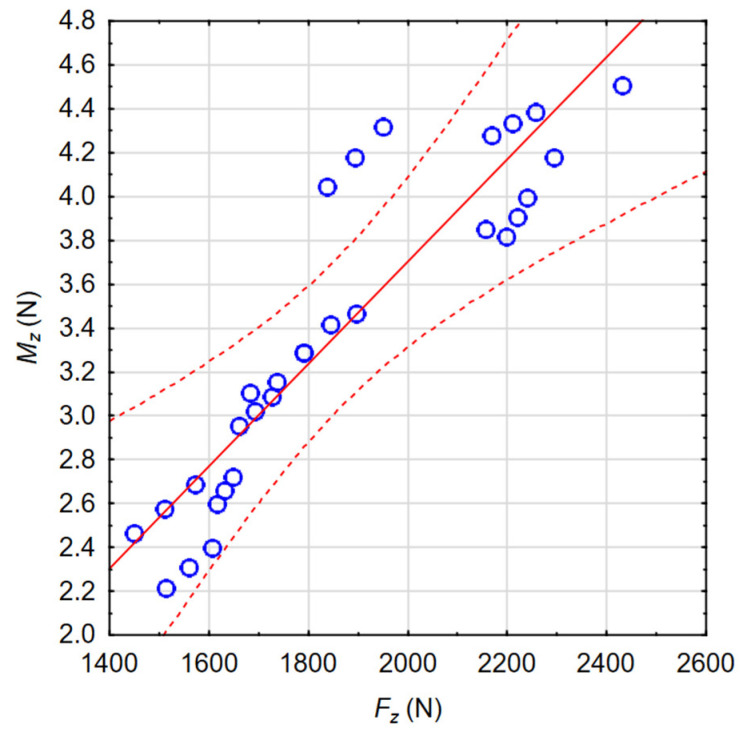
Relationships between the recorded values of the axial force *F_z_* and the torque *M_z_.*

Moreover, during the process of drilling holes in superalloy Inconel 718 material, correlations were observed between the recorded values of axial force and torque. The relationship between *F_z_* and *M_z_* together with the confidence interval for *α* = 0.05 is shown in Figure 21 (*R* = 0.91).

**Figure 22:**
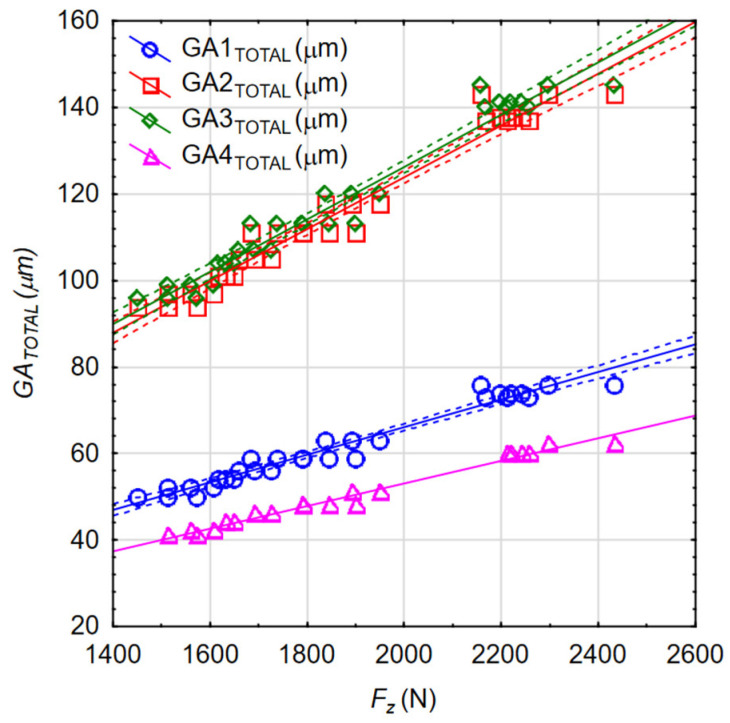
Relationships between the recorded values of the axial force *F_z_* and the engine case body deflections *GA_TOTAL_*.

Due to the correlated values of *v_c_, f, F_z_*, and *M_z_*, there was also a correlation between these values and the engine body deflection. A graph showing the relationships between the recorded values of the *F_z_* force and the body deflections *GA1_TOTAL_*, *GA2_TOTAL_*, *GA3_TOTAL_*, and *GA4_TOTAL_* together with the confidence intervals for *α* = 0.05 is shown in Figure 22. For all these cases, the Pearson correlation coefficients were R = 0.98. In the case of the analysis of the influence of the *F_z_* force on the deflection values *HA1_RADIAL_*, *HA2_RADIAL_*, *HA3_RADIAL_*, and *HA4_RADIAL_*, the Pearson correlation coefficients were in the range R = 0.97–0.98. Such values of this coefficient indicate a strong relationship between the analyzed factors. Also, the analysis between the values of the moment *M_z_* and the global deflection *GA_TOTAL_* and the radial displacement of the drilled hole axis *HA_RADIAL_* provides the ability to obtain values of the R coefficients in the ranges of 0.89–0.90 and 0.88–0.90, respectively.

**Figure 23:**
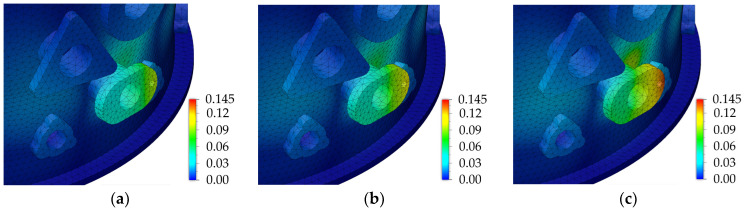
The influence of feed per revolution *f* at a constant cutting speed *v_c_* = 25 m/min on the *GA3_TOTAL_* engine body deflection: (**a**) *f* = 0.065 mm/rev, *F_z_* = 1521 N, and *M_z_* = 2.39 Nm; (**b**) *f* = 0.1 mm/rev, *F_z_* = 1807 N, and *M_z_* = 3.37 Nm; and (**c**) *f* = 0.135 mm/rev, *F_z_* = 2351 N, and *M_z_* = 4.35 Nm.

Taking into account the above correlations, for the designed engine case body, there is a relationship between the values of the applied cutting parameters, the deflection of the engine case body, and the radial displacement of the drilled hole axis. This is a consequence of the influence of the used cutting parameters on the values of axial force and torque, which affects the deformation of the engine body. The effects of increasing the feed per revolution *f* at a constant cutting speed of *v_c_* = 25 m/min on the body deformations occurring during the drilling of adapter No. 3 holes are shown in Figure 23a–c. In order to better illustrate the analyzed impact, only parts of the geometry are presented.

**Figure 24:**
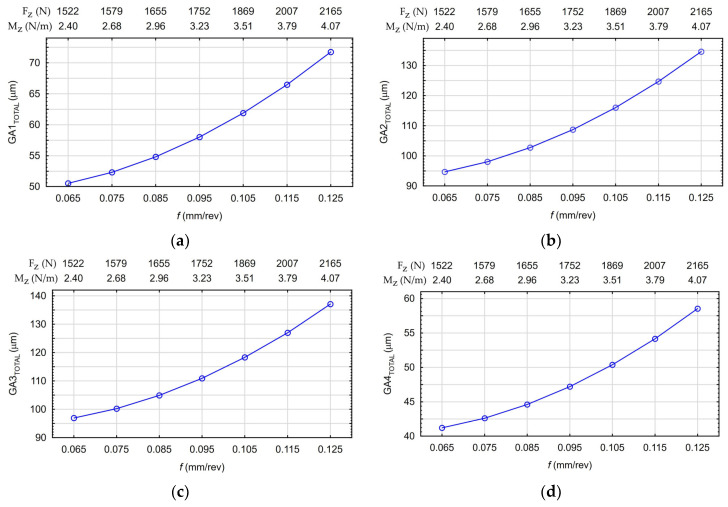
The influences of the feed per revolution *f* (at the constant *v_c_* = 25 m/min), axial force *F_z_*, and the torque *M_z_* values on the *GA_TOTAL_* body deflection occurring during the drilling of individual holes: (**a**) *GA1_TOTAL_*, (**b**) *GA2_TOTAL_*, (**c**) *GA3_TOTAL_*, and (**d**) *GA4_TOTAL_*.

Figure 24a–d show the calculations made by the created models on the influences of the feed per revolution *f* (at the constant *v_c_* = 25 m/min), axial force *F_z_*, and the torque *M_z_* values on the *GA_TOTAL_* body deflection occurring during the drilling of individual holes.

**Figure 25:**
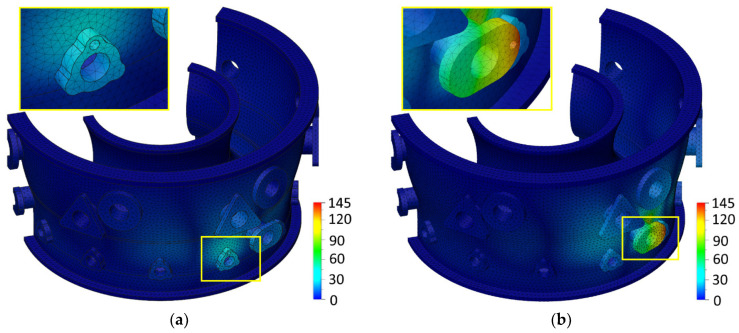
The calculated minimum and maximum deflection *GA_TOTAL_*: (**a**) *GA4_TOTAL_* and *v_c_* = 25 m/min, and *f* = 0.065 mm/rev; (**b**) *GA3_TOTAL_* and *v_c_* = 25 m/min, and *f* = 0.135 mm/rev.

The analysis of the results showed that the *GA_TOTAL_* deflection values, depending on the applied cutting parameters and the hole tooling adapter, ranged from 41 µm to 145 µm. The body deformations for the smallest and largest calculated deflection values occurring during the drilling of holes with the tested values of cutting parameters *v_c_* and *f* are shown in Figure 25a,b.

**Figure 26:**
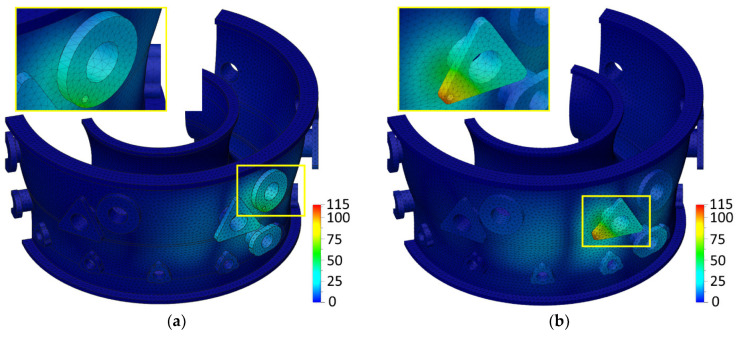

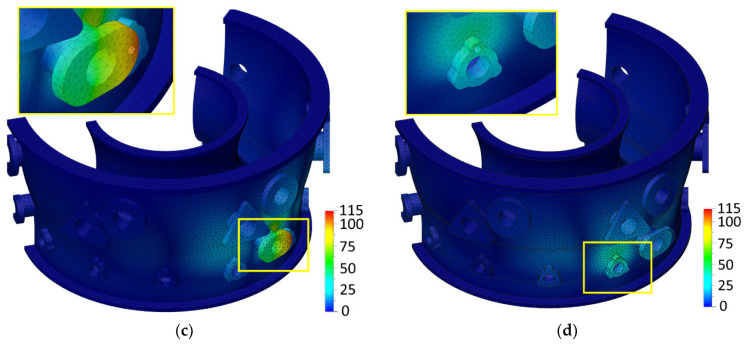
Observed engine case body deflection *GA_TOTAL_* at constant *v_c_* = 25 m/min and *f* = 0.1 mm/rev: (**a**) *GA1_TOTAL_*; (**b**) *GA2_TOTAL_*; (**c**) *GA3_TOTAL_*; and (**d**) *GA4_TOTAL_*.

On the other hand, a smaller effect of hole machining on the body deflection was observed for the tooling adapters located in the upper and lower areas of the engine case body. In these places, the stiffness of the designed body was the highest because it is located near the flanges on which the body is fixed during drilling. Figure 26a–d show the calculated body deflections for the machining of individual tooling adapters together with the middle values adopted for cutting parameters (*v_c_* = 25 m/min, *f* = 0.1 mm/rev).

## 5. Conclusions

This article evaluates the influence of the hole drilling process’s cutting speed *v_c_* and feed *f* on the geometry deflection and the displacement of the hole axis values. The analysis was performed for aircraft engine body tooling adapters during the hole drilling process. Effective hole machining poses many challenges due to the small wall thickness of the engine body and the material from which the body is most often made—Inconel 718. The research was conducted in two stages. In the first stage, using the Response Surface Method and based on the conducted experimental studies, mathematical models of the influence of cutting speed and feed on the axial force and torque were created. In the second stage of the research, using the Finite Element Method, the calculations of the maximum body deflection and the displacement in the radial direction of the axis of the currently drilled hole were made. For this purpose, the created geometric model of the engine case body was loaded with forces and moments recorded during the first stage of the research. The machining of four types of engine tooling adapters was considered. As a result of the research and analysis, the following main conclusions were formulated:

1. During the drilling process in the Inconel 718 alloy, relatively high values of the axial component of the axial cutting force exceeding 2200 N were recorded. The values of the recorded torques were characterized by relatively low values in the range of up to 4.33 Nm.
2. Increasing the feed has a strong adverse effect on the values of the axial force and torque. Increasing the cutting speed causes a beneficial reduction in the values of the axial force and torque.
3. In the analyzed drilling process, there was a strong positive correlation (*R* = 0.91) between the recorded values of the axial force and torque. There was also a strong positive correlation (*R* > 0.97) between the values of the engine case body deflections and the cutting parameters used in the drilling process.
4. The greatest geometry deflections for the engine case body occur in the direction of the applied axial cutting force outside the area of the hole being made. Loading the engine case body with the analyzed forces and moments in each analyzed case causes deformations in the elastic range.
5. The observed values of the displacements of the hole axes in the radial direction, depending on the cutting parameters and the tool adapter hole being machined, ranged from 13 µm to 68 µm. By taking into account these displacements in the process of designing the machining of tooling adapter holes, it was possible to minimize the positioning and shape errors of the holes.

Based on the proposed research method, it is possible to determine the body deflection and displacement of the hole axes resulting from the use of a set of drilling process parameters. The influence of the drilling process parameters on the cutting force and torque values for the tested material and tool was also determined. Based on the presented methodology, it was possible to scale to other cutting tools, machined materials, and body geometry. These results can be implemented in the process of designing the hole machining for the aircraft engine body. Based on the determined displacements of the hole axes, their positions can be corrected so that, as a result of machining, better accuracy of the arrangement of accessories and adapter holes for the aircraft engine body can be obtained. Values for cutting parameters that ensure that plastic deformation, causing body damage, which does not occur in the workpiece, can also be determined. Using the proposed solution, integration between the techniques of conducting the experiment, performing calculations using FEM, and designing the machining process is also achieved.

The proposed research methodology, due to deliberate action, does not take into account the making of a very high-cost real engine body and, therefore, does not take into account the verification studies of the calculations made using the finite element method. Thus, the accuracy of these calculations can only be estimated. In the case of industrial design, an expensive-to-fabricate real model may be available, so the verification tests can be taken into account.

The Response Surface Method used in research has allowed the development of mathematical models to describe the analyzed phenomena. However, other methods can also be used here. In the future, the methodology described in this paper can be adapted using the Levenberg–Marquardt method or other methods based on Artificial Intelligence.

## Figures and Tables

**Table 1:** The chemical composition of Inconel 718 (wt. %) [24].

Ni	Cr	Fe	Nb	Mo	Ti	Al	Co	Mn	C	Si	P
50–55	17–21	Balance	4.75–5.5	2.8–3.3	0.65–1.15	0.2–0.8	<1	<0.35	<0.08	<0.35	<0.015

**Table 2:** Research plan with assumed levels and values of process parameters.

Test No.	Case	*v_c_* (m/min)	*f* (mm/rev)
1	9 (C) *	25.0	0.1
2	5	17.93	0.1
3	2	20.0	0.125
4	7	25.0	0.065
5	6	32.07	0.1
6	3	30.0	0.075
7	1	20.0	0.075
8	8	25.0	0.135
9	4	30.0	0.125
10	10 (C) *	25.0	0.1

* The symbol (C) indicates the tests at the center of the research plan.

**Table 3:** Parameters of the applied material model.

Material	
Material Type	Isotropic Material
Mass Density	8600 (kg/m^3^)
Mechanical	
Young’s Modulus	185,200 N/mm^2^ (MPa)
Poisson’s Ratio	0.30
Strength	
Yield Strength	772 N/mm^2^ (MPa)

**Table 4:** The ANOVA analysis results for *Fz* and *Mz*.

	*F_z_*					*M_z_*				
Source	Seq SS	DF	MS	F-Value	*p*-Value	Seq SS	DF	MS	F-Value	*p*-Value
*v_c_*	**45,326**	**1**	**45,326**	**7.62**	**0.011**	**2.200**	**1**	**2.1996**	**38.93**	**0.000**
*v_c_* ^2^	3037	1	3037	0.51	0.482	0.150	1	0.1503	2.66	0.117
*f*	**2,065,163**	**1**	**2,065,163**	**346.97**	**0.000**	**11.525**	**1**	**11.5252**	**203.99**	**0.000**
*f* ^2^	**61,381**	**1**	**61,381**	**10.31**	**0.004**	0.000	1	0.0000	0.00	0.994
*v_c_·f*	4742	1	4742	0.80	0.381	0.004	1	0.0043	0.08	0.784
Residual Error	136,897	24	5952			1.299	24	0.0565		
Total	2,319,149	29				15.227	29			

Seq SS—the sum of squares due to the source. DF—the degrees of freedom in the source. MS—the mean sum of squares due to the source. F-Value—the variance of the group means. *p*-Value—the probability that the difference between groups is due to chance. Bold font indicates important factors with a statistically significant influence on the measurement results

## Data Availability

The original contributions presented in this study are included in the article. Further inquiries can be directed to the corresponding author.
